# Discovery and Protein Modeling Studies of Novel Compound Mutations Causing Resistance to Multiple Tyrosine Kinase Inhibitors in Chronic Myeloid Leukemia

**DOI:** 10.31557/APJCP.2020.21.12.3517

**Published:** 2020-12

**Authors:** Zafar Iqbal, Muhammad Absar, Amer Mahmood, Aamer Aleem, Mudassar Iqbal, Abid Jameel, Tanveer Akhtar, Sajjad Karim, Mahmood Rasool, Zeenat Mirza, Muhammad Khalid, Afia Muhammad Akram, Muhammad Farooq Sabar, Ahmad M Khalid, Khalid Aljarrah, Janhangir Iqbal, Muhammad Khalid, Ijaz H Shah, Nawaf Alanazi

**Affiliations:** 1 *Hematology Oncology and Pharmacogenetics Engineering Sciences (HOPES) Group, Health Sciences Research Laboratories, Department of Zoology, University of the Punjab, Lahore, & University of Education, Lahore, Pakistan. *; 2 *Department of Anatomy, College of Medicine and King Khalid University Hospital, King Saud University, Riyadh, Saudi Arabia. *; 3 *Hematology/Oncology Division, Department of Medicine, College of Medicine and King Khalid University Hospital, King Saud University, Riyadh, Saudi Arabia. *; 4 *Foreign Faculty, Asian Medical Institute, Kant City, National Surgical Centre, Bishkek, Kyrgyzstan, and Higher Education Commission Program in “Hematology Oncology and Pharmacogenetics Engineering Sciences (HOPES)”, Kyrgyzstan. *; 5 *Post-Graduate Medical Institute, Hayatabad Medical Complex, Peshawar, Pakistan.*; 6 *Center of Excellence in Genomic Medicine Research & Department of Medical Laboratory Technology, Faculty of Applied Medical Sciences, King Abdulaziz University, Jeddah, Saudi Arabia. *; 7 *Blood Bank, Peshawar, Pakistan. *; 8 *Department of Zoology, Division of Science and Technology, University of Education, Township, Lahore, Pakistan. *; 9 *Centre for Advanced Molecular Biology, University of the Punjab, Lahore, Pakistan. *; 10 *Departments of Biotechnology and Genomic Medicine, University of Sialkot, Pakistan.*; 11 *College of Applied Medical Sciences, King Saud Bin Abdulaziz University for Health Sciences (KSAU-HS)/ KAIMRC/SSBMT, National Guards Health Affairs, Al-Ahsa, Kingdom of Saudi Arabia. *; 12 *Jordan University of Science and Technology, Irbid, Jordan. *; 13 *National Guard Health Affairs, King Abdullah International Medical Research Centre (KAIMRC), Al-Ahsa, Saudi Arabia. *; 14 *Allied Hospital, Punjab Medical College & Sahil Hospital, Faisalabad, Pakistan. *

**Keywords:** Nilotinib, BCR-ABL mutation, chronic myeloid leukemia, tyrosine kinase inhibitors

## Abstract

**Objective:**

BCR-ABL fusion oncogene is the hallmark of chronic myeloid leukemia (CML), causing genomic instability which leads to accumulation of mutations in *BCR-ABL* as well as other genes. *BCR-ABL* mutations are the cause of tyrosine kinase inhibitors (TKIs) resistance in CML. Recently, compound BCR-ABL mutations have been reported to resist all FDA approved TKIs. Therefore, finding novel compound *BCR-ABL* mutations can help and clinically manage CML. Therefore, our objective was to find out novel drug-resistant compound BCR-ABL mutations in CML and carry out their protein modelling studies.

**Methodology:**

Peripheral blood samples were collected from ten imatinib resistant CML patients receiving nilotinib treatment. BCR-ABL transcript mutations were investigated by employing capillary sequencing. Patient follow-up was carried out using European LeukemiaNet guidelines. Protein modeling studies were carried out for new compound mutations using PyMol to see the effects of mutations at structural level.

**Results:**

A novel compound mutation (K245N mutation along with G250W mutation) and previously known T351I utation was detected in two of the nilotinib resistance CML patients respectively while in the rest of 8 nilotinib responders, no resistant mutations were detected. Protein modelling studies indicated changes in BCR-ABL mutant protein which may have negatively impacted its binding with nilotinib leading to drug resistance.

**Conclusion:**

We report a novel nilotinib resistant BCR-ABL compound mutation (K245N along with G250W mutation) which impacts structural modification in BCR-ABL mutant protein leading to drug resistance. As compound mutations pose a new threat by causing resistance to all FDA approved tyrosine kinase inhibitors in BCR-ABL+ leukemias, our study opens a new direction for in vitro characterization of novel BCR-ABL compound mutations and their resistant to second generation and third generation TKIs.

## Introduction

Chronic myeloid leukemia, a myeloproliferative disorder, is reported in roughly 15% of newly diagnosed leukemia in adults (Izzo et al., 2019). BCR-ABL, an oncoprotein central to the pathogenesis of CML, is fusion product of Abelson murine leukemia (ABL) and breakpoint cluster region (*BCR*) genes located on chromosome 9 and 22 respectively (Rowley, 1973). BCR-ABL, a tyrosine kinase, is constitutively expressed to promote growth and replication (Himburg et al., 2019). Genomic instability in CML is well established that is primarily caused by BCR-ABL itself, and it leads to acquisition of further mutations in BCR-ABL itself as well as other genes with the clinical course of the disease (Slupianek et al., 2011; Wu et al., 2018).

Imatinib mesylate (Gleevec^®^ USA; Glivec-Europe/Australia; formerly STI571) is a BCR-ABL TKI which has intensely improved the treatment outcomes in chronic-phase CML patients (CML-CP). The drug inhibits BCR-ABL by binding to an inactive conformation of the kinase, preventing signal transduction and cellular proliferation (Druker et al., 2001; Druker et al., 1996). Regardless of the efficacy of imatinib in patients with Ph-positive (Ph+) CML-CP, approximately 15% of patients display resistance to imatinib or relapse after an initial therapeutic response and thus have less favorable long-term outcomes (Hochhaus et al., 2009). A simple mechanism of resistance to TKI treatment is owing to mutations in the kinase domain of BCR-ABL. Such mutations have been reported in 40% to 60% of imatinib-resistant patients (Jabbour et al., 2006). More than 90 distinct mutations in BCR-ABL have been recognized, each conferring variable degrees of resistance to imatinib treatment (Soverini et al., 2010).

Nilotinib, a small rationally designed TKI by Tasigna; Novartis Pharmaceuticals Corporation, is good inhibitor of BCR-ABL and is approved for imatinib-resistant CML. in vitro study shown wide inhibitory activity of nilotinib against most of the imatinib-resistant mutants of BCR-ABL (Weisberg et al., 2006). Previous data have demonstrated that imatinib-resistant patients with CML-CP or CML-accelerated phase (AP) treated with nilotinib had high and durable rates of response for all mutations except T315I, Y253H, E255K/V, and F359C/V (Hughes et al., 2009; Kantarjian et al., 2011). In last few years, compound BCR-ABL mutations (multiple BCR-ABL mutations in a single molecule) have posed a new threat by causing resistance to all FDA approved TKIs including T315I-mutation specific drug ponatinib (Kang et al., 2018). Studies related to compound BCR-ABL mutations can help in early determination of drug resistance, to find out complex mechanisms of multi-drug resistance and help in designing new drugs for multiple / compound mutant CML cases (Hochhaus et al., 2020; Soverini et al., 2020). Here we report a novel compound BCR-ABL mutation causing multi-TKI resistance CML as well as structural BCR-ABL changes induced by this mutant associated with multiple kinase inhibitor resistance. 

## Materials and Methods

*Patients and blood sample collection*

A total of 10 CML imatinib resistant patients who had received nilotinib treatment for at least 12 months or more, from Allied Hospital Faisalabad, Pakistan, were included in this study. Number of patients was low as very few patients could afford nilotinib, a very expensive drug. Three ml blood was taken in labeled EDTA tubes per manufacturer’s instructions. Blood was processed within 24 hours to ensure minimum degradation of mRNA, otherwise stored at -40°C. Randomization was stratified according to the Sokal risk score at the time of diagnosis. This study was approved by the local ethics committee or review board and was conducted in accordance with the Declaration of Helsinki with written consent form each patient.

*Clinical history and patient’s data*

A complete clinical history since the diagnosis of CML were collected in patient data forms and required permission and consent were taken from hospital authorities and participating patient. Patients were routinely followed-up to record hematologic and cytogenetic response on monthly basis. We followed European Leukemia Net guidelines for categorizing CML patient samples into drug sensitive or resistant on the basis of their response to therapy (Baccarani et al., 2014).

*RNA extraction from blood samples*

RNA was extracted from patient blood using protocols described previously (Chomczynski et al., 1987; Liedtke et al., 1994). All RNA extraction steps were carried out on crushed ice to evade degradation of nucleic acid. Isolated RNA was stored immediately at -40˚C till further processing.

*Qualitative analysis of RNA*

Quality of RNA was assessed by performing native agarose gel electrophoresis (Biocom and PowerPac Basic, BIO-RAD) and RNA was observed as two discrete fluorescent bands (28S and 18S) (UVP PhotoDoc-ItTM Imaging System). 

*Complementary DNA synthesis*

cDNA was synthesized by reverse transcription of RNA previously described protocol with slight variations (Thermo scientific™, USA) (van Dongen et al., 1999). 

*cDNA integrity check*

Integrity of cDNA was assessed by amplification of housekeeping gene, glyceraldehyde 3-phosphate dehydrogenase (*GAPDH*) using forward: 5ˊ-ACCACAGTCCATGCCATCA-3ˊ and reverse: 5ˊ-TCCACCACCCTGTTGCTGTA-3ˊ primers (Thermo Scientific™, USA and Applied Biosystems 2720 thermal cycler) (Asad et al., 2012). 

*Nested reverse transcription PCR (nRT-PCR)*

Amplification of BCR-ABL fusion oncogene was performed by nRT-PCR (Applied Biosystems 2720 thermal cycler) using long range enzyme (Thermo ScientificTM, USA). For comparing CML patients (n =10) with control, we amplified the kinase domain of *ABL* gene from healthy population (n = 20). Primer sequences and PCR reaction conditions were followed from Willis et al., 2005 (Willis et al., 2005), with slight variations (Table 1).

Purification (Gel extraction) of amplified PCR products

To avoid any contamination of non-specific DNA fragments during sequencing, PCR products were purified from agarose gel (Quick gel extraction Kit, Invitrogen) and visualized (UVP PhotoDoc-ItTM Imaging System). Needed PCR products of specific size were carefully excised, and purified PCR products were stored at -20˚C for sequencing or further use.

*Direct Sequencing analysis of PCR amplicons*

To detect any likely point mutation in ABL-kinase domain of CML patients, we directly sequenced the amplified products by Sanger’s method using 5ˊ-TGGTTCATCATCATTCAACGG-3ˊ and 5ˊ-GGACATGCCATAGGTAGCA-3ˊ primer pair (ABI-3130XL and ABI-3730 genetic analyzers, Applied Biosystems, USA) (Willis et al., 2005).

*Analysis of sequencing data*

To find out any polymorphic variations in our population, the ABL kinase domain DNA sequences of healthy individuals were aligned against the reference sequence (NCBI’s GenBank Accession Number: M14752.1). Polymorphic variations were excluded while detecting point mutations in CML patient’s sequences (Geneious R7). Entire kinase domain sequence including P-loop, C-Helix, SH2 contact and activation loop was explored to identify any probable nucleotide substitutions responsible for altered response to imatinib treatment.

*Statistical analysis*

Fisher’s exact test (Chi-square test) was done for comparing patient’s response (sensitivity or resistance) towards therapy and mutation status among different clinical categories or predictors of CML patients (IBM SPSS version 19, Chicago, IL, USA.).

*Bioinformatics Analysis*

Structural bioinformatics analysis was done for the BCR-ABL protein for structurally validating our findings. Crystallographically determined human ABL kinase protein structure in complex with nilotinib was retrieved from worldwide archive of structural data of biological macromolecules, RCSB’s PDB (PDB ID: 3cs9) and viewed using Delano Scientific’s PyMOL (DeLano, 2002). Further modeling studies were done to find out structural changes responsible for drug resistance. Modeling studies for structure and function prediction of the mutant structure was done using I-Tasser server with alignment generated using MUSTER threading program (Laskowski and Swindells, 2011). Detailed structural visualization and comparative alignment was done for the wild type and mutant modeled using PyMOL. LigPlot+ v.1.4.5 program (EBI, Cambridge, UK) was used to check and schematically plot the polar and hydrophobic interactions between the protein BCR- ABL kinase and the inhibitor nilotinib (Cortes et al., 2012). The 3-D structure of the protein-ligand complex was given as an input in PDB format and the software displayed their interacting residues and bonds.

## Results

*Patient demographics, clinical and lab oratory findings*

Peripheral-blood samples were collected from imatinib resistant CML patients aged ≥18 years who were receiving nilotinib (400 mg twice daily) for at least 12 months. Clinical characteristics of CML patients, wherein all were in chronic phase CML have been summarized in Table 2. The response to nilotinib was determined by examining the clinical end points: complete hematological response (CHR), and complete cytogenetic response (CCyR, n=2). Number of male patients was more than females with male: female ratio of 8:2. Splenomegaly (80%) was the most common symptom followed by fever, weight loss (40%), fatigue and hepatomegaly (20%). The mean age of patients in the present study was 37.2 years (age range: 28-50 years). White cell count ranging within normal values (0.5-1.0 x 10^9^/L) was observed in 2 patient, while 8 individuals had more. Eight patients had platelet count between 100- 450 x 10^9^/L, while 2 had more than 450 x 109/L. Six patients had blast cells less than 5% and four patients had their hemoglobin level < 10g/dl). Sokal risk scores were low of six, intermediate for two and high for two CML patients. Hematological response was complete for eight and not for two, while cytogenetic response was four, four and two for complete, partial and minor respectively. No statistically significant association (p=<0.05) was observed in the different parameters of the studied subjects.

*RNA extraction and Nested RT-PCR amplification of BCR-ABL kinase domain*

**Figure 1:**
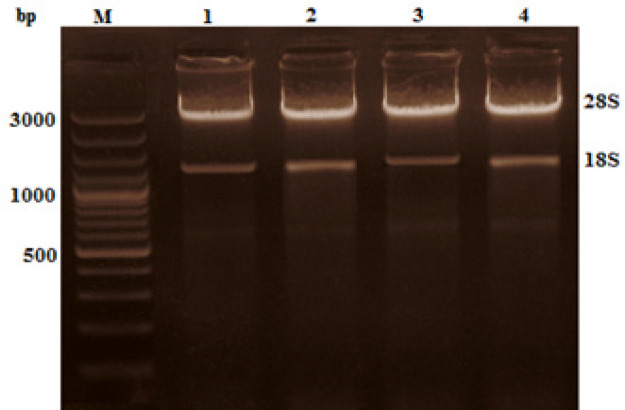
Visualization of Total RNA Isolated from Blood Samples of CML Patients on 1.5% Agarose Gel. Intact 28S and 18S RNA were observed. bp= Base pairs, M= DNA marker

**Figure 2:**
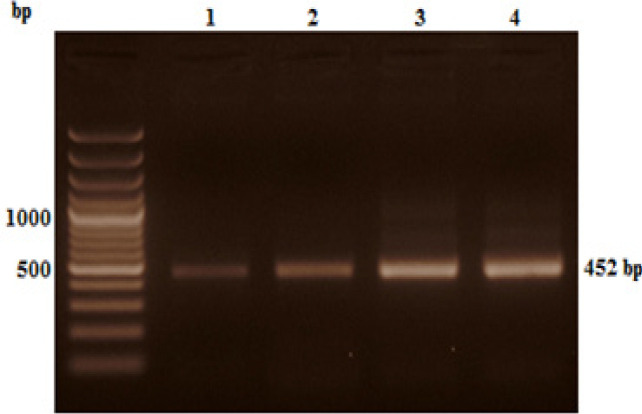
Visualization of GAPDH Amplification to Check cDNA Quality and Integrity on 1.5% Agarose Gel; bp= Base pairs, M= DNA marker

**Figure 3:**
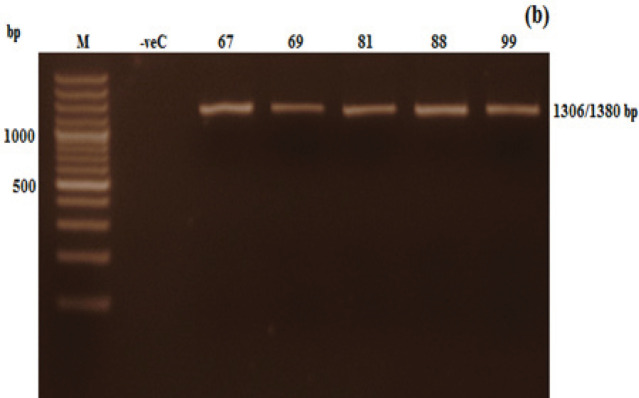
Visualization of Nested PCR (round 2) Products of Nilotinib Sensitive Wild Type (a) and nilotinib sensitive mutant (b) CML patients on 1% agarose gel. bp= Base pairs, M= DNA marker, -veC= Negative control

The total RNA isolated from blood samples of CML patients was run on 1.5% agarose gel to check the integrity of extracted RNA (Figure 1). The PCR quality of cDNA was evaluated by running the amplification product of *GAPDH* housekeeping gene on 1.5% agarose gel (Figure 2). The nested PCR of the *BCR-AB*L gene was successfully amplified in all samples at the end of second round nested RT-PCR. Nested PCR products of nilotinib sensitive wild type (a) nilotinib sensitive mutant (b) CML patients were run on 1% agarose gel. As CML patients had two common variants of fusion oncogene, the resulting products in different samples were 1306 bp (b2a2) and 1380 bp (b3a2) in size (Figure 3). 

*Direct DNA Sequencing*

**Figure 4:**
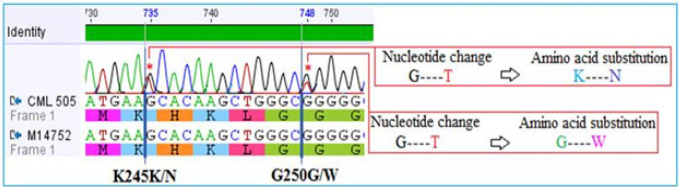
Electropherogram Showing Compound Mutations, Including a Novel BCR-ABL Mutation Associated with Primary Nilotinib Resistance in CML Patient

Direct sequencing analysis of the amplified PCR products was performed to detect point mutations in the ABL-kinase domain of CML cases. The electropherogram showing compound mutations, including a novel BCR-ABL mutation associated with primary nilotinib resistance in CML patient (Figure 4). The nucleotide changes and their subsequent amino acid substitutions are shown by blue longitudinal indicator in each sample along with the nucleotide number. NCBI GenBank accession number: M14752.1 is the reference sequence.

**Figure 5:**
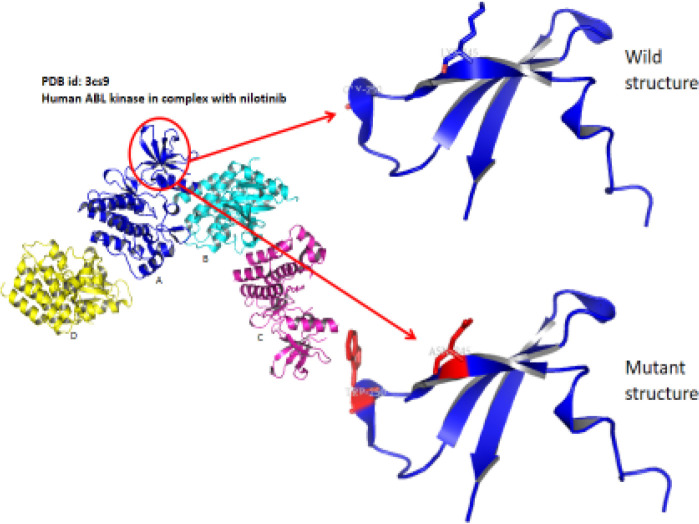
Mutant Structure was Made Using PyMol

**Figure 6:**
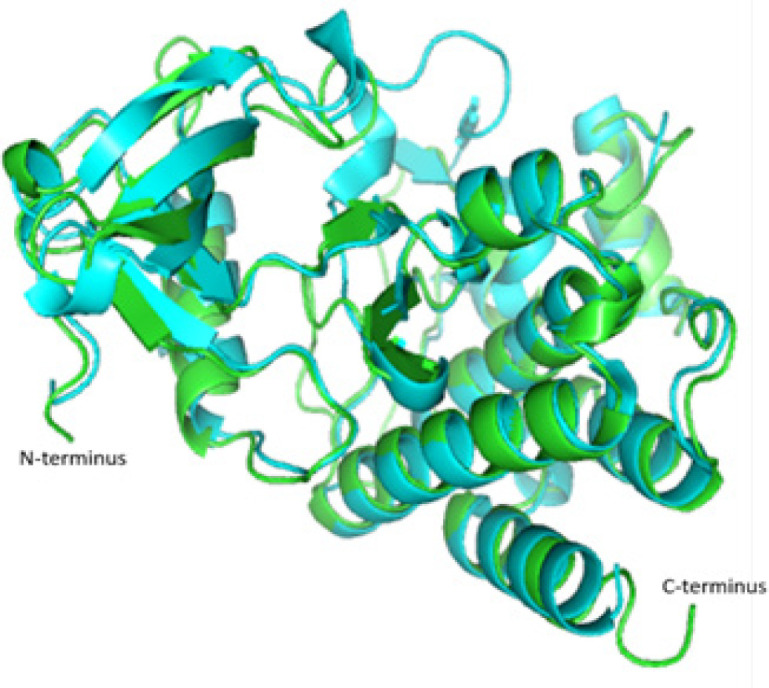
Structural Alignment Done Using PyMol of Wild (PDB id: 3cs9) and Mutant (Modeled) Protein. In the overlapped structure, green represents mutant and cyan represents the wild type

**Figure 7:**
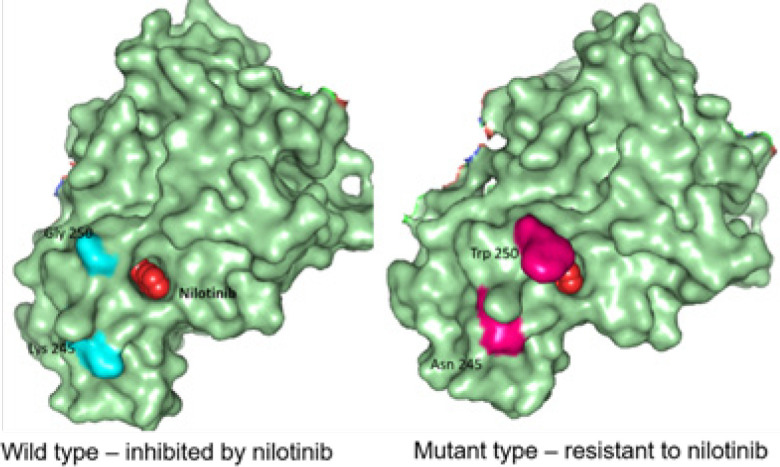
Comparative Structure Analysis Highlighting the Hydrophobic Pocket with the Mutation Induced Topographical Difference in the Two Cases

**Figure 8:**
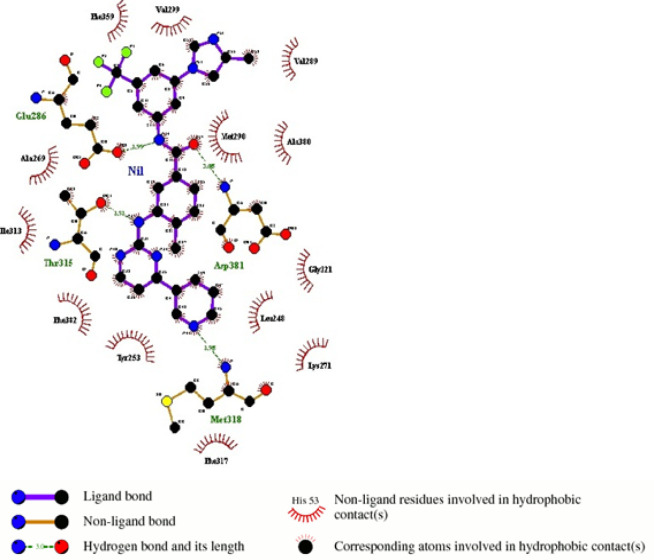
Schematic 2D Binding Mode Representation with the Ligand Nilotinib as Generated by Ligplot+ v.1.4.5

Molecular Structural Studies: Molecular modeling were done to find out structural changes induced in wild type (Lys245, Gly250) mutated to mutant type (Asp245, Trp250) responsible for conferring drug resistance. Our residues of interest lies within the glycine-rich phosphate loop (P-loop) traversing from 242-256 constitute (Figure 5, 6, and 7). Ligplot analysis was carried to check the schematic 2-D molecular interactions between kinase protein and the bound ligand nilotinib (Figure 8). The primarily interacting residues were Glu286, Thr315, Met318 and Asp381.

**Table 1:** Primer Sequences for Amplification of BCR/ABL-kinase Domain by Nested RT-PCR

Nested PCR	Primer name	Primer Sequence (5′-3′)
Round 1	B2A forward	5′-TTCAGAAGCTTCTCCCTGACAT-3′
	ABL4065 reverse	5′-CCTTCTCTAGCAGCTCATACACCTG -3′
Round 2	BCR F4 forward	5′-ACAGCATTCCGCTGACCATCAATA-3′
	U396 reverse	5′-GCCATAGGTAGCAATTTCCC-3′

**Table 2:** Clinical Characteristics of CML Patients at Time of Disease Diagnosis and Patients Response (Hematologic and Cytogenetic) at the Time of Blood Sample Collection

Patient Characteristics N (%)			
Clinical and laboratory characteristics at diagnosis	Gender	Male	8 (80)
		Female	2 (20)
		Spleen enlargement	8 (80)
		Fever	4 (40)
	Symptoms	Hepatomegaly	2 (20)
		Fatigue	2 (20)
		Weight loss	4 (40)
	White cell count (x 10^9^/L)	10-May	2 (20)
		> 10	8 (80)
	Platelet count (x 10^9^/L)	100-450	8 (80)
		>450	2 (20)
	Blast cells in peripheral blood	Less than 5%	6 (60)
		More than 5%	4 (40)
	Hemoglobin in peripheral blood	<10g /dl	4 (40)
		>10g /dl	6 (60)
	Sokal risk score	Low	6 (60)
		Intermediate	2 (20)
		High	2 (20)
Patient’s response at the time of blood sample collection	Phase at the time of blood collection	Chronic	10 (100)
		Accelerated	0
		Blast	0
	Hematological Response (HR)	Complete	8 (80)
		No	2 (20)
	Cytogenetic Response (CyR)	Complete	4 (40)
		Partial	4 (40)
		Minor	2 (20)

## Discussion

In the current study all CP-CML patients who were receiving nilotinib (≥ 12 months) second line TKI therapy after imatinib resistance were included and investigated for any mutations related to nilotinib resistance. Male to female ratio was 4:1, respectively. In previous literature incidence of CML with male-to-female ratio of 1.1:1 to 1.74:1 had been reported (Aziz et al., 2010; Jacobs et al., 2018). The altered ratio in present study is owing to small number of available patients on nilotinib therapy. Enlargement of spleen was the most commonly observed symptom in the patients during clinical examination (80%). This finding is in concordance with the conclusions that massive splenomegaly is often associated with CML (Efficace et al., 2011). The incidence of other signs and symptoms; including fever, hepatomegaly and fatigue etc. varies in literature and is stated to be 40-64.4%, 21.7-100% and 22-100%, respectively (Aguayo et al., 2008; Karimi et al., 2008). 

Among the patients included in the present studies, 80% showed a massive over-production of WBCs ranging between 12-670 x 10^9^/L at the time of diagnosis. In literature, leukocytosis (>10 x 10^9^/L) has been stated between 40.7--77.3% of patients with CML [29, 35]. The platelet counts ranged between 105-694 x 10^9^/L. Only one patient had platelet count >450 x 10^9^/L i.e. 694 x 10^9^/L (20%) which is in agreement to the reported range of platelet count in CML patents (10.2-21.4%) (Jabbour et al., 2011; Jacobs et al., 2018). Hemoglobin level lower 10g/dl was evident in 2 patients (40%) In cancer patient’s low Hb level has been reported in range of 15-59.3% of CML patients (Jacobs et al., 2018; Jabbour et al., 2017).

The monitoring of cytogenetic response (CyR) in CML has a prognostic value and CCyR achievement has been linked with prolonged survival (Druker et al., 2006). In the present study, patients exhibiting CCyR, partial CyR and minor CyR was 40%, 40% and 20%, respectively. Similarly, HR is also important in CML response monitoring. In the present study, CP-CML patients showed CHR to be 80%. In literature, HR has been reported to be 90% 53 and 93.75% (Gupta and Prasad, 2007; Hochhaus and Kantarjian, 2013). 

In the present study, 1 out 5 CP-CML patients treated with nilotinib 400 mg twice daily, compound mutations, including a novel *K245N* mutation and *G250W *mutation in BCR-ABL domain were detected. The presence of this compound mutations most likely have contributed to nilotinib resistance in this patient. This is the first report of such novel mutation occurring in *BCR-ABL*. The nilotinib resistant patient progressed to accelerated phase (AP), however, mutations alone are not a cause of disease progress to advanced stage or treatment failure and alternative mechanisms may be involved (Melo and Barnes, 2007). Furthermore, we cannot confirm whether treatment-emergent mutation in our case is solely a driver mutation and cause of disease progress to advance stage. The remaining patients (n=4) of nilotinib responders did not show any mutations. *G250E* (P-loop) mutation has been reported in CML patients treated with either imatinib or nilotinib (Melo and Barnes, 2007). 

Although BCR–ABL inhibitor based treatment helps majority of CML patients to lead normal life till treatment continue but unfortunately few patients i.e. 20-30% develop TKI resistance, either primary or secondary and are prone to progress toward the AP or BP of the disease (Kang et al., 2006). *BCR-ABL* mutations are the major background players in manifestation of resistance to all FDA approved TKIs including imatinib, dasatinib and nilotinib (Hochhaus et al., 2020; Baccarani et al., 2014). According to European or North American clinical guidelines, detection of mutations is a vital during drug resistance or drug switching, because nature of mutation determine the resistance or sensitivity for different drugs. So in case of resistance or unsatisfactory response to *BCR-ABL* inhibitors, mutational information can help in deciding better alternative TKI. Till date four main mutational regions for TKIs resistance have been reported: P-loop, SH3 contact (3) SH2 contact and (4) A-loop (Kang et al., 2006; Druker etal., 2001).

Nilotinib, a second-generation inhibitor, has greater potency and specificity (approximately 20 fold) for wild type BCR-ABL than imatinib (Blay et al., 2011). It binds to ABL tyrosine kinase and disrupt the ATP-phosphate-binding site causing inhibition of catalytic activity of enzyme. Other than *T315I* mutation, it inhibits all imatinib-resistant mutants (Hamad et al., 2013). Clinically, nilotinib based treatment is recommended by ELN for all newly diagnosed Ph+ CML-CP and imatinib intolerant Ph+ CML in CP or AP patients. Better understanding of reasons and mechanism of resistance will improve the treatment strategy(Hamad et al., 2013). Nilotinib is good suppressor of overall resistance causing *BCR–ABL* mutations (Marchesi et al., 2013), and found to sensitive against D816V mutant variant, which is resistant to imatinib (Weisberg et al., 2007). Blast crisis stage of patients need more attention as about 40–90% of *BCR–ABL* mutation in imatinib resistant patients detected here (Soverini et al., 2020).

Coexistence of two or more mutations in the same *BCR-ABL mRNA*, is termed as a “compound mutation”. Patients harboring compound mutations like V299L, *T315A, F317L/V/I/C17 *(Soverini et al., 2006; Burgess et al., 2005) were found to be less sensitive to dasatinib therapy and nilotinib treatment failure has been reported in several studies in patients with compound mutations like* E255K/V,Y253H, F359V/C/I *[Hughes et al., 2009; Soverini et al., 2009; Khorashad et al., 2013).

Khorashad et al., (2013) analyzed *BCR-ABL* kinase domain mutations in 1,700 samples by direct DNA sequencing and found that 11.4% CML patient samples harbored ≥2 mutations. In total, 30 different compound mutations were observed, with the most frequent mutation being T315I among all compound mutants. These mutations existed in two forms: compound or polyclonal. Compound mutations (multiple mutations in the same *BCR-ABL* molecule) accounted for 70.2% and 29.8% were polyclonal mutations (mutations occurring in multiple BCR-ABL molecules) . Therefore, the distinction between compound versus single mutations is clinically important because it may influence the selection of the most suitable TKI to overcome resistance. Several compound mutations have been shown to confer resistance to ponatinib and this is likely to apply to other third-line TKIs as well (O’Hare et al., 2009; Yang et al., 2014).

Yang et al., (2014), evaluated the effect of nilotinib on two CML imatinib resistant patients with *V299L* mutation. Nilotinib treatment successfully turned *V299L* mutation of both cases into negative. Acquired *BCR-ABL* mutations related to nilotinib treatment in patients with CML was initially available from two meeting abstracts (Hughes et al., 2007). The first of these described *F349V, E255K/V, E355G, G250E, M244V *and *T315I*. The second report described *Y253F, E255V, T315I*, and *F359V *as emerging mutations. Many mutations developing in response to nilotinib have now been described in literature allowing improved interpretation. These mutations include: *T315I, Y253C, Y253H, E255V, L248V, K285N, E282K, E255K, F359C, F359V, K247N, W430L, T406I, E255R, A380S, Y253F, Q252H, L387F, F311V, L273F, H375P, H396P, E292K, D276G, E275K, L387M, G250E, E431G, V379I, Q252H, F317L, F311L, N297T, E355A, F317V, H396R, G250A, E355G, F486S, V289L, M244V, D325N, M388L, F317C, G250V, M237I, S348L, E255D, E281K, M351T, E453K, E344G, E459Q, F311I, A433T, F349V, E292V, L384M, E274K, L370P, M388I, E450K, S438C, E459G, E459K* (Khorashad et al., 2013; Hughes et al., 2007), and *F359I/V, E279K, L248L/V* (Ursan et al., 2015).

A systematic literature search was performed by Ursan et al., (2015) between January 1966 and May 2013, regarding use of nilotinib as first line TKI in CML patients. A single study of nilotinib was found in this search as first-line treatment in CML that included 61 patients, in whom the mutation incidence was 3.3% (95% CI = 0.0%-7.7%). In these patients (n=61), *E255K* and *F359C* mutations were reported and experienced disease progression (Branford et al., 2009). Clinically relevant mutations to second-generation inhibitors were* F317L/I/C/V, V299L*, and *T315A* for dasatinib; *Y253H, E255K/V, *and* F359V/C* for nilotinib; and* T315I* for both *TKIs* (Goodrich, 2014).

A 3^rd^ generation molecule such as ponatinib, has been approved by FDA and can inhibit majority of mutations in kinase domain. However, it’s unlikely that ponatinib will be used for treatment because of drug dosing issue (Yang et al., 2014). Additionally, ponatinib had been found to be vulnerable to couple of compound mutations like *G250E/T315I *and *E255V/T315I*. These* BCR-ABL1 *compound mutants causing high-level resistance against ponatinib is next significant challenge to 2^nd^ and 3^rd^ generation TKIs in CML patients (Marchesi, 2013; Eide et al., 2011; Azam et al., 2009). 

Protein structure modeling of Human ABL kinase - nilotinib complex was done to find out structural changes responsible for resistance. The residues spanning from 242-256 constitute the glycine-rich phosphate loop (P-loop) that facilitates the transphosphorylation reaction. Residues of present interest (245 and 250) are not directly involved in binding with the drug. However, may contribute to the development of resistance against nilotinib in the mutated protein. Substitution mutation (glycine, the smallest amino acid to the bulkiest aromatic tryptophan) will potentially hinder the smooth entry of the drug into the binding cavity. Additionally, the mutation in the surface residue from basic Lys to acidic Asn might also interfere with inhibitor binding. In past, BCR-ABL protein modelling studies have led to unravel mechanisms of TKI resistance in CML and to design novel drugs to treat drug-resistant CML (Ozkan et al., 2018; Altıntop et al., 2017). We hope that reported compound *BCR-ABL* mutations and its protein modelling studies will help unravel complex mechanism of drug resistance and to design more effective drugs for CML. Further studies on compound *BCR-ABL *mutations, specifically resistance to 2^nd ^and 3^rd^ generation TKI resistance, by employing genomic techniques will lead to better clinical management of recent challenges in CML treatment (Hochhaus et al., 2020; Soverini et al., 2020). 

In summary, novel compound mutations resulted due to innate genomic instability in *BCR-ABL+ CML* cells leading to nilotinib resistance have been reported in our studies. Protein modelling studies showed that mutations caused structural changes in BCR-ABL protein which may have impacted binding of drug to the BCR-ABL target and led to nilotinib resistance. It is important to perform mutational analysis prior to drug switching and distinction between compound versus single mutation because it may influence the clinical selection of the most suitable TKI to overcome resistance. Studies on novel compound *BCR-ABL* mutations associated with multiple TKI resistance will help understand mechanism of drug resistance in CML in more depth and will lead to better drug resistance management strategies to improve CML survival, possibly leading to a potential cure 
